# Phenotypic and genotypic characterization of *Staphylococcus aureus*, including MRSA and PVL-positive isolates, in Egyptian fruit bats

**DOI:** 10.1186/s12917-026-05591-9

**Published:** 2026-06-04

**Authors:** Toka A. Allam, Fatma Abdel-Kader, Mona Kadry

**Affiliations:** Department of Zoonoses, Faculty of Veterinary Medicine, Cairo University, PO Box 12211, Giza, Egypt

**Keywords:** Egypt, Bats, PVL, MRSA, MDR, *Staphylococcus aureus*

## Abstract

**Background:**

Bats are widely distributed mammals capable of harboring diverse pathogens, including antimicrobial-resistant bacteria. *Staphylococcus aureus* (*S. aureus*), particularly methicillin-resistant *S. aureus* (MRSA), poses a significant public health concern. This study aimed to investigate the occurrence, antimicrobial resistance profile, and presence of the Panton–Valentine leukocidin (*pvl*) virulence gene of *S. aureus* in Egyptian fruit bats.

**Methods:**

Fifty Egyptian fruit bats were captured using mist nets at both foraging and roosting sites and morphologically identified. A total of 150 samples (intestinal contents, oral swabs, and skin swabs) were collected for bacteriological examination. *S. aureus* isolates were confirmed by Gram staining, catalase test, and coagulase test. Genomic DNA was extracted for molecular identification using PCR targeting the 16S rRNA and *nuc* genes. Antimicrobial susceptibility testing was conducted using the disk diffusion method. PCR was additionally used to screen for methicillin resistance genes (*mecA *and *mecC*) and the *pvl* virulence gene.

**Results:**

At the sample level, 67 of 150 samples (44.7%) were confirmed as *S. aureus*, while 42 of 50 bats (84.0%) tested positive at the bat level. The highest prevalence was observed in skin swabs (56.0%), followed by intestinal contents (40.0%) and oral swabs (38.0%). Eighteen isolates (26.9%) were classified as MRSA, and five isolates (7.5%) were multidrug-resistant (MDR), all of which were also MRSA. PCR analysis detected the *mecA* gene in 7 of the 18 (38.9%) MRSA isolates, while the *mecC* gene was not detected in any sample (0.0%). The *pvl* gene was identified in 6 of 67 isolates (9.0%). MRSA isolates exhibited diverse antimicrobial resistance patterns, with 61.1% demonstrating a multiple antimicrobial resistance index (MARI) value ≥ 0.2.

**Conclusion:**

This study provides evidence of *S. aureus* carriage in Egyptian fruit bats, including MRSA and PVL-positive isolates, some of which were multidrug-resistant. These findings suggest that bats may serve as ecological carriers of antimicrobial-resistant and virulent bacteria, highlighting the importance of wildlife surveillance within a One Health framework.

## Introduction

Bats (order Chiroptera) are widely distributed mammals found on every continent except the polar regions, representing more than 20% of all mammalian species [1]. Their biological characteristics, including powered flight, long-distance movement, colonial roosting, and ecological adaptability, contribute to their ecological success and support their role as reservoirs of diverse infectious agents [2]. In recent years, bats have received increasing attention due to their involvement in zoonotic disease ecology, particularly under conditions of increasing human–wildlife interaction driven by habitat disturbance [3, 4].

Bats harbor a wide range of zoonotic pathogens, including bacteria, fungi, protozoa, and viruses [5]. While viral pathogens have been extensively studied, bacterial communities and antimicrobial resistance (AMR) in bats have received comparatively less attention [6–8]. Fruit bats (*Rousettus* spp.) are widely distributed in tropical and subtropical regions and often inhabit caves, buildings, and urban environments. Their feeding behavior and mobility expose them to diverse microbial populations, including antimicrobial-resistant bacteria, highlighting their potential role in environmental microbial circulation [9, 10].

Antimicrobial resistance is a major global public health challenge, projected to cause up to 10 million deaths annually by 2050 if no effective interventions are implemented [11]. The detection of antimicrobial-resistant bacteria in wildlife, where antibiotics are not directly used, is particularly concerning and suggests anthropogenic environmental contamination [12]. Antibiotic residues in soil, water, and food can select for resistant strains that may subsequently be carried by wildlife, making bats potential ecological sentinels that reflect the impact of human activities through the presence of antimicrobial-resistant organisms in their habitats [12].

Reports of multidrug-resistant bacteria in wildlife from areas with high human activity underscore the role of environmental exposure in the spread of AMR, including MRSA. Several studies have documented resistance patterns among both Gram-positive and Gram-negative bacteria isolated from bats worldwide, supporting their potential role in the environmental circulation of AMR [13–15].

*Staphylococcus aureus* is an important opportunistic pathogen responsible for a wide spectrum of diseases, including skin and soft tissue infections, bacteremia, endocarditis, pneumonia, and foodborne illness [16]. Beyond humans, it has been isolated from numerous domestic animals and wildlife species [17]. One of the major public health concerns to emerge in recent decades is MRSA, due to its resistance to most β-lactam antibiotics, mediated by the staphylococcal cassette chromosome mec (SCCmec), which carries the *mecA* and *mecC* genes encoding altered penicillin-binding proteins [18]. MRSA frequently exhibits resistance to multiple antimicrobial classes, including aminoglycosides, macrolides, fluoroquinolones, chloramphenicol, and tetracyclines [19], resulting in multidrug-resistant phenotypes that complicate treatment and infection control [20].

Although MRSA was historically associated with healthcare settings, distinct community-associated and livestock-associated strains have emerged, and wildlife has increasingly been recognized as part of its epidemiology [21–23]. Evidence suggests that methicillin resistance may have originated in wildlife species, such as hedgehogs, even prior to the clinical use of antibiotics [24], underscoring the importance of incorporating wildlife into AMR surveillance frameworks.

In addition to antimicrobial resistance, *S. aureus* possesses a wide array of virulence determinants, including Panton–Valentine leukocidin (PVL) toxin, a cytotoxin associated with severe skin and soft tissue infections and necrotizing pneumonia [25, 26]. PVL-positive strains are of particular concern due to their association with severe clinical outcomes, including high mortality in cases of PVL-mediated pneumonia [27].

In Egypt, MRSA prevalence among clinical *S. aureus* isolates is relatively high compared to other regions [28]. Given the ecological mobility of bats and their frequent contact with human-modified environments, they may harbor antimicrobial-resistant and virulent *S. aureus* strains [29, 30]. Therefore, investigating the occurrence of *S. aureus*, MRSA, multidrug-resistant (MDR), and PVL-positive isolates in bats is important for better understanding their role in bacterial ecology and antimicrobial resistance circulation.

## Materials and methods

### Bat capture, morphological identification, and sample collection

Fifty fruit bats were captured from multiple roosting and foraging sites in Menofia Governorate, Egypt, including caves, agricultural areas, and tree roosts. Capture was carried out during December 2024 –February 2025. All captured bats were apparently healthy at the time of capture, with no visible signs of injury or disease. The study included 36.0 females and 14.0 males, and all specimens were confirmed to be adults based on forearm length measurements (≥ 130.0 mm) and complete epiphyseal fusion of the wing-finger joints, assessed by gentle palpation at capture.

All procedures were conducted in accordance with institutional and international animal welfare guidelines. Euthanasia was necessary to allow for the aseptic collection of intestinal contents, a terminal procedure required to fulfill the study’s microbiological objectives. Bats were humanely euthanized by isoflurane inhalation until total loss of reflexes and respiratory arrest, followed by cervical dislocation to confirm death. All experimental protocols were approved by the Institutional Animal Care and Use Committee (IACUC) of the Faculty of Veterinary Medicine, Cairo University, Egypt (Approval No. Vet CU110520251177), and the study is reported in accordance with the ARRIVE guidelines.

At the time of euthanasia, body weights ranged from 100.0 to 130.0 g. Species identification was conducted based on external morphological characteristics in accordance with the taxonomic keys described by Dietz [31] and Monadjem et al. [32]. Diagnostic features included large eyes and simple ears lacking both tragus and antitragus, which are characteristic of the family Pteropodidae. The presence of claws on both the first and second digits further supported this classification. Morphometric measurements, including wingspan, forearm length, and tail length, were recorded using a mechanical caliper. Collectively, these morphological criteria confirmed that all examined specimens belonged to the genus *Rousettus*.

Three types of biological samples were aseptically collected from each bat: oral swabs, skin swabs, and intestinal contents, yielding a total of 150 samples (50 per sample type). Oral swabs were obtained by firmly rotating a sterile cotton swab within the oral cavity, covering the tongue, gingiva, and buccal mucosa. Skin swabs were collected from hairless or sparsely haired regions of the body surface, specifically the ventral aspect of the wing membrane (patagium), the axillary region, and the uropatagium.

For intestinal content collection, the abdominal cavity was opened under aseptic conditions, and the gastrointestinal tract was carefully excised. The intestinal lumen was opened longitudinally, and luminal contents were gently scraped to ensure representative sampling.

### Isolation and identification of *S. aureus*

Each sample was enriched overnight in Brain Heart Infusion (BHI) broth (HiMedia, India) supplemented with 6.5% NaCl and incubated at 37 °C for 24 h. The enriched cultures were then streaked onto Mannitol Salt Agar (MSA) (HiMedia, India) and incubated aerobically at 37 °C for 24 h. From each positive sample, three colonies showing characteristic golden-yellow pigmentation and mannitol fermentation on MSA were selected, subcultured to obtain pure isolates, and examined for colony morphology. Preliminary identification of *S. aureus* was confirmed by Gram staining (Gram-positive cocci in clusters), catalase test, and coagulase test, according to Markey et al. [33] and Mahon et al. [34].

To ensure biosafety and prevent laboratory contamination, standard precautions were strictly followed, including the use of personal protective equipment (PPE), adherence to hand hygiene practices, routine disinfection of work surfaces and instruments, and proper disposal of biological waste.

### Extraction of genomic DNA

Genomic DNA was extracted from each presumptively identified *S. aureus* isolate using the standard boiling method, as previously described by Dimitrakopoulou et al. [35] and Yahya Ahmed et al. [36]. DNA concentration and purity were assessed using a NanoDrop spectrophotometer. The extracted DNA was stored at − 20 °C until further use.

### Molecular identification of the *Staphylococcus* genus and *S. aureus* species

The *Staphylococcus* genus was confirmed by PCR targeting the 16S rRNA gene, while *S. aureus* species identification was verified by amplification of the *nuc* gene, according to McClure et al. [37]. PCR reactions were performed in a total volume of 25 µl, containing 3 µl of template DNA, 12.5 µl of 2x amaR OnePCR™ master mix (GeneDireX, Inc., USA), 0.5 µl of each primer (10 pmol/µl; Metabion, Germany), and PCR-grade water to a final volume of 25 µl. PCR amplicons were resolved on a 1.5% agarose gel and visualized under UV illumination. Primer sequences and amplification conditions are provided in Table 1.

**Table 1:** Primers and PCR cycling conditions for housekeeping, resistance, and virulence genes of *Staphylococcus aureus*

Gene (amplicon size, bp)	Primer sequence	Cycling condition	Reference
*16 S* rRNA (756)	F: AACTCTGTTATTAGG GAAGAACA R: CCACCTTCCTCCGGT TTGTCACC	94 °C ,5 min; 30 cycles (94 °C, 1 min; 50 °C, 1 min; 72 °C, 1 min), 72 °C ,5 min	[37]
*nuc* *(279)*	F: GCGATTGATGGTGATACGGTT R: AGCCAAGCCTTGACGAACTAAAGC	95 °C ,5 min; 30 cycles (95 °C, 30 s; 55 °C, 30 s; 72 °C, 1 min), 72 °C ,10 min	[37]
*mec A* (776)	F: *TGGCTCAGGTACTGCTATCCAC* R: *AGTTCTGCAGTACCGGATTTGC*	94 °C ,5 min; 30 cycles (94 °C, 30 s; 60 °C, 30 s; 72 °C, 30s), 72 °C ,10 min	[41]
*mec C* (304)	F: GCTCCTAATGCTAATGCA R: TAAGCAATAATGACTACC	94 °C ,5 min; 30 cycles (94 °C, 30 s; 50 °C, 30 s; 72 °C, 30s), 72 °C ,10 min	[41]
*pvl* (433)	F: ATCATTAGGTAAAATGTCTGGACATGATCCA R: GCATCAAGTGTATTGGATAGCAAAAGC	94 °C ,3 min; 35 cycles (94 °C, 1 min; 50 °C, 45 s ; 72 °C, 45 s), 72 °C ,5 min	[42]

“F, forward primer; R, reverse primer; bp, base pairs; s, seconds; min, minutes”

### Antimicrobial Susceptibility Testing (AST)

Antimicrobial susceptibility testing was performed using the Kirby–Bauer disk diffusion method in accordance with the guidelines of the Clinical and Laboratory Standards Institute (CLSI) [38]. A total of 14 commercially available antimicrobial disks (HiMedia, India), representing ten antimicrobial classes, were tested on Mueller–Hinton agar (MHA). The antibiotics tested included β-lactams (cefoxitin (FOX) 30 µg and methicillin (MET) 5 µg), aminoglycosides (amikacin (AK) 30 µg and gentamicin (GEN) 10 µg), macrolides (erythromycin (E) 30 µg and azithromycin (AZM) 15 µg), lincosamides (clindamycin (CD) 2 µg), sulfonamides (trimethoprim/sulfamethoxazole (COT) 1.25/23.75 µg), phenicols (chloramphenicol (C) 30 µg), ansamycins (rifampicin (RIF) 5 µg), nitrofurans (nitrofurantoin (NIT) 300 µg), tetracyclines (doxycycline (DOX) 30 µg), and fluoroquinolones (ciprofloxacin (CIP) 5 µg and levofloxacin (LE) 5 µg). *S. aureus* ATCC 25,923 was used as a quality control strain.

Antimicrobial susceptibility results were interpreted according to CLSI breakpoints. MRSA isolates were phenotypically identified based on resistance to cefoxitin. MDR was defined as resistance to at least one antimicrobial agent in three or more antimicrobial classes [39]. The multiple antibiotic resistance index (MARI) was calculated by dividing the number of antibiotics to which an isolate was resistant by the total number of antibiotics tested. A MARI value ≥ 0.2 indicates that the isolates originated from a high-risk source of contamination with frequent or extensive antibiotic exposure [40].

### PCR assay for detection of methicillin resistance and virulence genes in *S. aureus* isolates

Isolates exhibiting resistance to cefoxitin were further analyzed by PCR to confirm the presence of methicillin resistance-encoding genes, including *mecA* and *mecC*, as described by Cuny et al. [41]. To assess virulence, the presence of the Panton–Valentine leukocidin (*pvl*) gene was determined using the primers and amplification conditions reported by McClure et al. [42].

A negative control containing all PCR components except template DNA (substituted with nuclease-free water) was included, while *S. aureus* ATCC 700,699 was used as the positive control. Each PCR reaction was prepared in a total volume of 25 µl, including 3 µl template DNA, 12.5 µl 2× amaR OnePCR™ master mix (GeneDireX, USA), 0.5 µl of forward and reverse primers (10 pmol/µl; Metabion, Germany), and sterile PCR-grade water. Amplification products were analyzed using 1.5% agarose gel electrophoresis and detected under UV light. Primer sequences and amplification conditions are presented in Table 1.

### Statistical analysis

Descriptive statistical analysis was performed to calculate the frequencies and percentages of isolates across different sample types. Prevalence estimates were determined at both the bat level and the sample level. The 95% confidence intervals (CIs) for all prevalence estimates were calculated using the Wilson score interval method. Antimicrobial resistance phenotypes, along with the presence or absence of resistance and virulence genes (*mecA*, *mecC*, and *pvl*) among MRSA isolates, were visualized using a heatmap generated in R software (version 4.4.0) with the *pheatmap* package (version 1.0.13). Isolates were grouped according to anatomical source to facilitate comparison of resistance and virulence profiles across sample types.

## Results

### Prevalence and antimicrobial resistance profiles of *Staphylococcus aureus* isolates in bats

At the bat level, *S. aureus* was detected in 42 out of 50 bats (84.0%; 95% CI: 71.5–91.7%), defined as positivity in at least one anatomical site. Among positive bats, 20 (40.0%; 95% CI: 27.6–54.0%) harbored *S. aureus* at multiple anatomical sites simultaneously, of which 5 bats (10.0%; 95% CI: 4.4–21.4%) were positive at all three sampled sites. At the sample level, 67 of 150 samples tested positive for *S. aureus*, yielding a sample-level prevalence of 44.7% (95% CI: 36.5–53.2%) (Table 2). Detection rates varied by anatomical site: *S. aureus* was most frequently isolated from skin swabs (28/50; 56.0%; 95% CI: 42.3–68.8%), followed by intestinal contents (20/50; 40.0%; 95% CI: 27.6–54.0%) and oral swabs (19/50; 38.0%; 95% CI: 25.6–52.2%).

**Table 2:** Prevalence of *S. aureus*, MRSA, PVL-positive, and MDR isolates among different bat anatomical sites

Sample type (*n*)	*S. aureus *positive *n* (%) (95% CI)	MRSA *n* (% of *S. aureus*) (95% CI)	PVL-positive *n* (% of *S. aureus*) (95% CI)	MDR *n* (% of *S. aureus*) (95% CI)
Skin Swab (50)	28 (56%; 42.3–68.8%)	8 (28.6%; 14.9–47.5%)	2 (7.1%; 2.0–22.7%)	1 (3.6%; 0.6–17.8%)
Intestinal content (50)	20 (40%; 27.6–54.0%)	4 (20.0%; 8.1–41.6%)	2 (10.0%; 2.8–29.7%)	3 (15.0%; 5.2–36.0%)
Oral swab (50)	19 (38%; 25.6–52.2%)	6 (31.6%; 15.4–53.5%)	2 (10.5%; 3.0–31.4%)	1 (5.3%; 0.9–24.6%)
Total (150)	67 (44.7%; 36.5–53.2%)	18 (26.9%; 17.4–38.6%)	6 (9.0%; 4.2–18.3%)	5 (7.5%; 3.2–16.5%)

“MRSA, methicillin-resistant *S. aureus*; MDR, multidrug-resistant; PVL, Panton–Valentine leukocidin; n, number of isolates”

All isolates were confirmed through a sequential identification workflow: Gram staining revealed Gram-positive cocci in clusters, biochemical testing confirmed positive catalase and coagulase reactions, and molecular analysis yielded positive results for both the *nuc* and 16 S rRNA genes, providing definitive species-level identification.

A total of 18 out of 67 isolates (26.9%; 95% CI: 17.4–38.6%) were classified as MRSA, with the highest prevalence observed among oral swab isolates (6/19; 31.6%; 95% CI: 15.4–53.5%), followed by skin swab isolates (8/28; 28.6%; 95% CI: 14.9–47.5%) and intestinal content isolates (4/20; 20.0%; 95% CI: 8.1–41.6%). MDR *S. aureus* strains were identified in 5 of the 67 isolates (7.5%; 95% CI: 3.2–16.5%), with the highest proportion observed in intestinal content isolates (3/20; 15.0%; 95% CI: 5.2–36.0%), compared with oral swab isolates (1/19; 5.3%; 95% CI: 0.9–24.6%) and skin swab isolates (1/28; 3.6%; 95% CI: 0.6–17.8%). Furthermore, 11 of the 67 isolates (16.4%; 95% CI: 9.3–27.6%) demonstrated a multiple antibiotic resistance index (MARI) value ≥ 0.2, indicating probable origin from environments with frequent or intensive antibiotic use.

### Phenotypic antimicrobial resistance of *Staphylococcus aureus* isolates

**Fig. 1:**
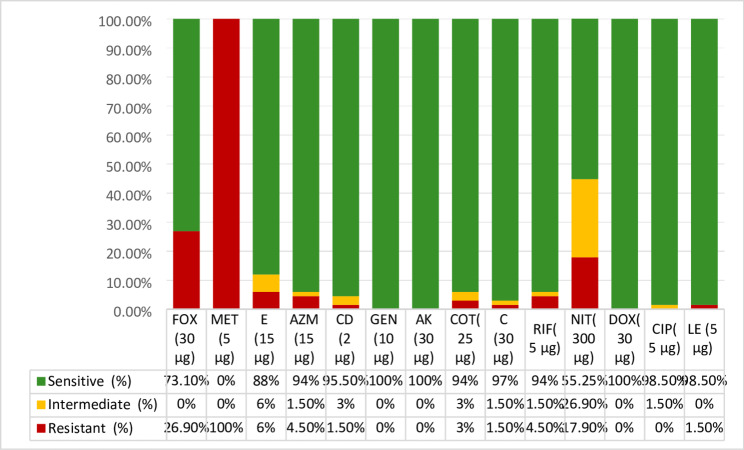
Percentage distribution of antimicrobial susceptibility patterns among the tested *S.aureus* isolates. FOX, cefoxitin (30 µg); MET, methicillin (5 µg); E, erythromycin (15 µg); AZM, azithromycin (15 µg); CD, clindamycin (2 µg); GEN, gentamicin (10 µg); AK, amikacin (30 µg); COT, trimethoprim–sulfamethoxazole (25 µg); C, chloramphenicol (30 µg); RIF, rifampicin (5 µg); NIT, nitrofurantoin (300 µg); DOX, doxycycline (30 µg); CIP, ciprofloxacin (5 µg); LE, levofloxacin (5 µg)

**Fig. 2:**
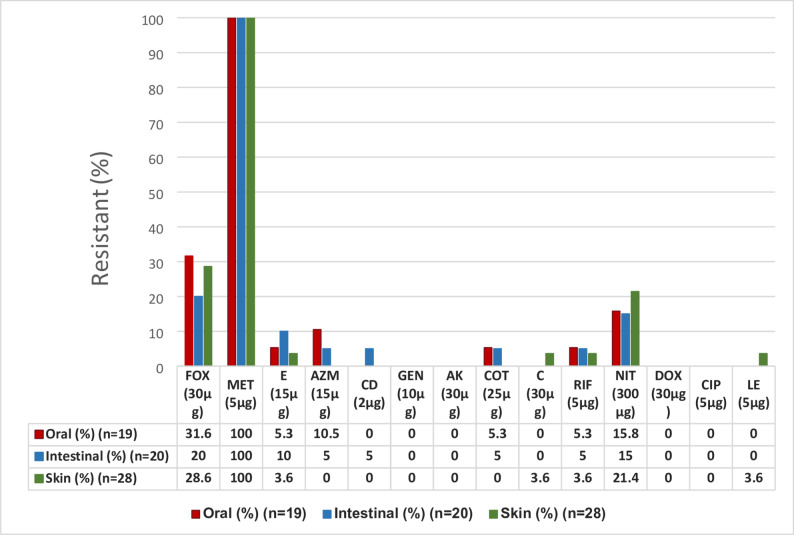
Resistance rates (%) of *S. aureus* isolates recovered from oral, intestinal, and skin samples of Egyptian fruit bats

The antimicrobial susceptibility profiles of the 67 *S. aureus* isolates recovered from bat skin swabs, intestinal contents, and oral swabs are summarized in Fig. 1. All isolates exhibited resistance to the methicillin disk; however, as methicillin disk testing is considered unreliable by current CLSI standards, MRSA classification was based solely on cefoxitin disk diffusion results. Accordingly, 18 isolates (26.9%; 95% CI: 17.4–38.6%) were confirmed as MRSA based on cefoxitin resistance. Moderate resistance was observed to nitrofurantoin (12/67; 17.9%; 95% CI: 10.4–28.6%), whereas resistance to other antimicrobial agents was low. All isolates were fully susceptible (100.0%) to aminoglycosides (gentamicin and amikacin) and tetracyclines (doxycycline), and high susceptibility was also observed for macrolides, clindamycin, trimethoprim–sulfamethoxazole, chloramphenicol, rifampicin, and fluoroquinolones (≥ 94.0%). When stratified by anatomical site (Fig. 2), cefoxitin resistance was highest in oral swab isolates, followed by skin swab and intestinal content isolates, whereas nitrofurantoin resistance was highest in skin swab isolates; resistance to other antimicrobial agents remained low and sporadic across all sample types.

### Genotypic characteristics of MRSA isolates recovered from bats

As presented in Table 3, PCR analysis targeting methicillin resistance genes (*mecA* and *mecC*) and the Panton–Valentine leukocidin (*pvl*) gene revealed that the *mecA* gene was detected in 7 of the 18 MRSA isolates (38.9%; 95% CI: 20.3–61.4%). The highest proportion of *mecA*-positive MRSA isolates was recovered from skin swabs (5/8; 62.5%; 95% CI: 30.6–86.3%), followed by oral swabs (2/6; 33.3%; 95% CI: 9.7–70.0%), whereas all intestinal content MRSA isolates (4/4; 100.0%) lacked the *mecA* gene. The *mecC* gene was not detected in any of the isolates examined (0.0%). The *pvl* gene was identified in 6 isolates, accounting for 9.0% of total *S. aureus* isolates and 33.3% of MRSA isolates.

**Table 3:** Distribution of *mecA* and *pvl* genes among MRSA isolates recovered from different bat anatomical sites (*n* = 18)

Sample type	MRSA (*n*)	*mecA* positive *n* (%) (95% CI)	*mecA* negative *n* (%) (95% CI)	PVL positive *n* (%) (95% CI)
Skin Swab	8	5 (62.5%; 30.6–86.3%)	3 (37.5%; 13.7–69.4%)	2 (25.0%; 7.1–59.1%)
Intestinal content	4	0 (0%)	4 (100%)	2 (50.0%; 15.0–85.0%)
Oral swab	6	2 (33.3%; 9.7–70.0%)	4 (66.7%; 30.0–90.3%)	2 (33.3%; 9.7–70.0%)
Total	18	7 (38.9%; 20.3–61.4%)	11 (61.1%; 38.6–79.7%)	6 (33.3%; 16.3–56.3%)

“MRSA, methicillin-resistant *S. aureus*; *mecA*, methicillin resistance-encoding genes; PVL, Panton–Valentine leucocidin”

### Phenotypic and genotypic resistance profiles of MRSA isolates recovered from bats

**Fig. 3:**
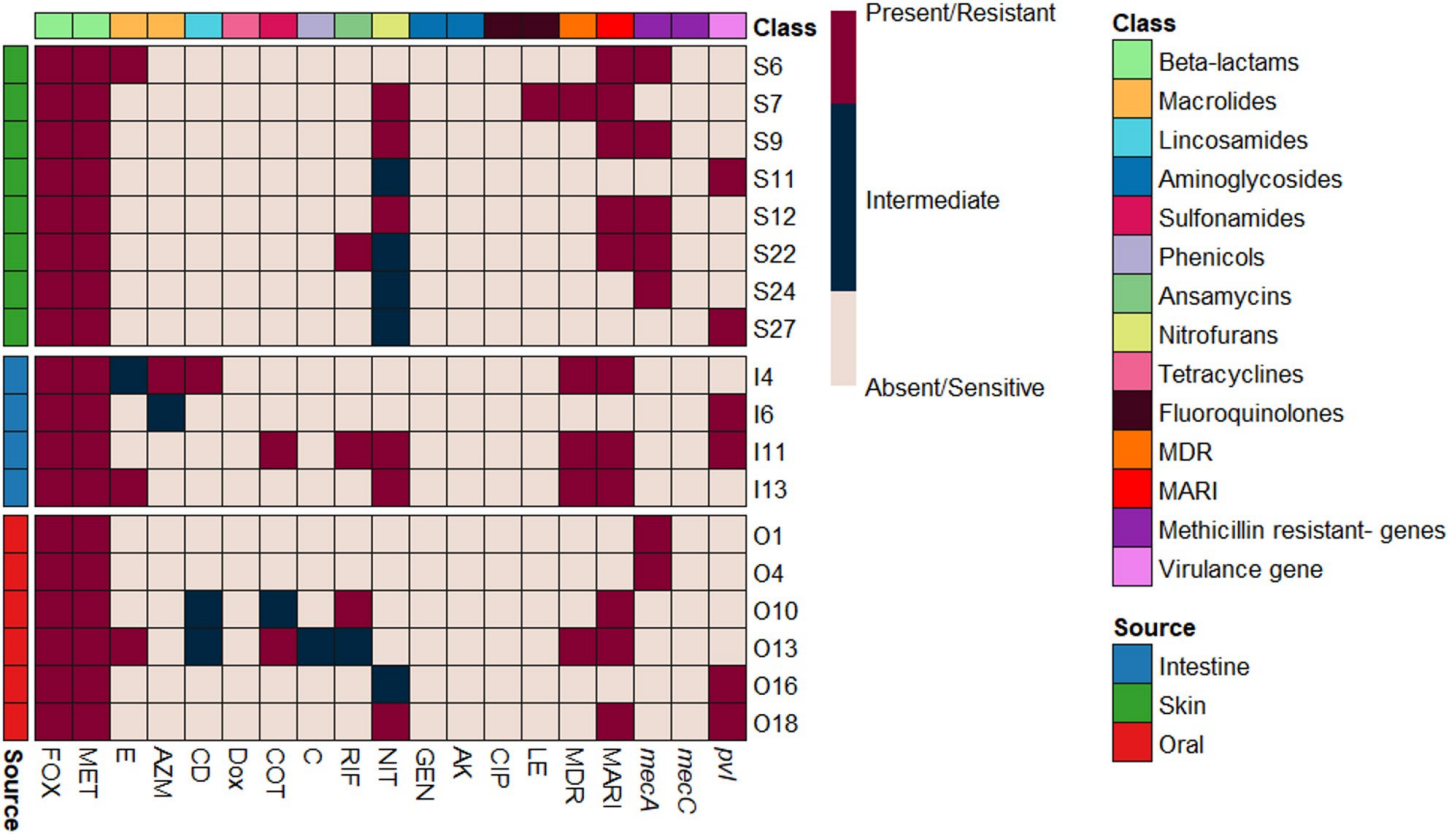
Heatmap clarified the antimicrobial resistance profiles of MRSA isolates against fourteen antibiotic discs. Phenotypic multidrug resistance (MDR) patterns and the Multiple Antibiotic Resistance Index (MARI) were assessed alongside the presence of methicillin-resistance encoding genes (*mecA* and *mecC*) and the virulence gene (*pvl*). Colors indicate MDR and MARI status (dark red, MDR-positive and MARI ≥ 0.2; Off-white, MDR-negative and MARI < 0.2), gene presence (dark red, present; Off-white absent), and antimicrobial susceptibility (resistant, dark red; intermediate, intermediate blue; sensitive, Off-white)

Analysis of the phenotypic resistance profiles of the 18 MRSA isolates revealed considerable variability across antimicrobial classes beyond cefoxitin and methicillin (Fig. 3). PVL-positive MRSA isolates were identified in both *mecA*-positive and *mecA*-negative backgrounds, demonstrating phenotypic heterogeneity among virulent isolates. Five isolates were identified as MDR-MRSA, exhibiting resistance to three or more antimicrobial classes, including rifampicin, macrolides, and trimethoprim–sulfamethoxazole. Furthermore, 11 of the 18 MRSA isolates (61.1%; 95% CI: 38.6–79.7%) demonstrated a MARI value ≥ 0.2, indicating origin from environments with high antibiotic selection pressure.

## Discussion

Bats are the only mammals capable of sustained flight, a characteristic that facilitates their wide geographic distribution and potential role in the dispersal of zoonotic pathogens [43]. While *S. aureus* is well-documented in humans, domestic animals, and livestock, data on its prevalence in wildlife remain limited [44]. In this study, *S. aureus* was detected in 42 out of 50 bats (84.0%) at the bat level and in 67 of 150 samples (44.7%) at the sample level. These findings are consistent with previous reports of *S. aureus* colonization in wild animals [45, 46], including bats [29, 47], and highlight the value of wildlife-based surveillance in understanding the broader epidemiology of this bacterium.

Among the isolates, the highest recovery rate was observed from skin swabs. This aligns with the findings of Lutz et al. [48], who reported that the microbial community associated with bat skin is significantly richer than those found in the gut or oral cavity. The skin likely provides favorable conditions, including moisture and temperature, that support bacterial survival [49]. Lower isolation rates were observed from intestinal contents and oral swabs. The distribution of isolates suggests that *S. aureus* is capable of adapting to various microenvironments within its host [50], and indicates the potential role of bat body surfaces as sites of bacterial carriage.

AMR is widely recognized as a major global public health concern [10]. Wildlife species are increasingly acknowledged as potential reservoirs of antibiotic-resistant bacteria and resistance genes [24]. Among them, bats represent a notable group that may harbor resistant strains, given their ecological adaptability and frequent habitation of environments near humans and domestic animals [51].

In the present study, 26.9% (18/67) of the *S. aureus* isolates recovered from bats were resistant to cefoxitin and classified as MRSA according to CLSI guidelines. This prevalence was higher than that reported by Carrillo Gaeta et al. [51] in bat skin samples from Brazil, but lower than the rate observed by Mairi et al. [52] in bat guano samples from Algeria. MRSA was detected across all sample types, with the highest proportion observed in oral swabs, followed by skin swabs and intestinal contents, suggesting that MRSA may colonize multiple anatomical sites within individual bats [53]. The detection of MRSA in wildlife is of growing interest, as numerous wild animal species, including mammals, birds, and rodents, have been documented as MRSA carriers [15, 22].

MDR *S. aureus* isolates were most frequently detected in intestinal content samples (15.0%), compared with skin and oral swabs. This may reflect greater exposure of the gastrointestinal tract to environmental sources of antimicrobial-resistant bacteria through contaminated food or water [54]. Five isolates were identified as both MDR and MRSA. Such MDR-MRSA strains are of public health concern, as resistance to multiple antimicrobial classes significantly limits treatment options and is associated with increased morbidity and mortality [55].

Furthermore, 11 of the 67 isolates (16.4%) exhibited a MARI value ≥ 0.2, indicating probable exposure to environments with frequent or intensive antibiotic use [40, 56]. This finding suggests that bats may inhabit anthropogenically impacted environments and may carry antimicrobial-resistant bacteria as a result. However, whether this reflects active transmission or passive environmental exposure requires further investigation.

The resistance profiles of bat-associated isolates revealed notable diversity across antimicrobial classes (Fig. 1). Resistance to macrolides was observed in seven isolates, encompassing both erythromycin and azithromycin. Additional resistance was detected to levofloxacin, chloramphenicol, and rifampicin, albeit at relatively low frequencies. These antimicrobials are critically important in both human and veterinary medicine, and the emergence of resistance, even at low levels, may have implications for therapeutic efficacy.

Resistance to clindamycin and trimethoprim–sulfamethoxazole was also detected; both agents are commonly used for the treatment of *S. aureus* and MRSA infections [57, 58]. Although the observed resistance rates were low, their presence warrants attention, as it may indicate the early emergence of resistance to agents frequently relied upon as alternative treatment options. Notably, resistance to nitrofurantoin was detected in 17.9% of isolates. Nitrofurantoin is widely used as a first-line treatment for urinary tract infections in humans, including those caused by multidrug-resistant pathogens [59]. The detection of nitrofurantoin-resistant isolates in bats may suggest indirect exposure to human-associated antimicrobial residues through shared environments.

In the present study, 7 of the 18 (38.9%) cefoxitin-resistant isolates carried the *mecA* gene, while none tested positive for *mecC* (0.0%). A discrepancy between phenotypic resistance and genotypic confirmation has been reported in previous studies involving both clinical and animal-derived isolates from Egypt and other regions [60–66], as well as in wildlife-associated *Staphylococci*, including bat isolates [51]. The *mecC* gene was not detected in any isolate, consistent with reports of its low prevalence in Egypt and globally [61, 67, 68]. Although *mecC* is a recognized *mecA* homolog associated with methicillin resistance in *Staphylococci*, its occurrence remains rare in these settings.

Cefoxitin resistance in the absence of both *mecA* and *mecC* was observed in 11 isolates. Several resistance mechanisms have been described in the literature, including alterations in penicillin-binding proteins, β-lactamase hyperproduction, and alternative *mec* homologs such as *mecB* or *mecD* [1, 69–71]; however, these were not investigated in the present study. Further studies using whole-genome sequencing or PBP2a detection assays are warranted to clarify the underlying resistance mechanisms in these isolates.

The pathogenicity of *S. aureus* is largely attributed to its ability to produce a wide range of virulence factors. Among these, Panton–Valentine leukocidin (PVL) is a pore-forming toxin known for its ability to destroy leukocytes and promote severe infections [25]. In the present study, the *pvl* gene was detected in 9.0% of total *S. aureus* isolates, indicating the presence of a virulence trait traditionally associated with community-associated MRSA (CA-MRSA) in humans [72]. All PVL-positive isolates were also resistant to cefoxitin, indicating the co-occurrence of virulence and methicillin resistance markers within the bat isolates examined. While such strains are associated with severe clinical manifestations in humans, including skin and soft tissue infections and, in rare cases, necrotizing pneumonia [26].

The detection of PVL-positive *S. aureus* has previously been reported in wildlife [73]. Olatimehin et al. [47] documented a markedly higher prevalence (78.6%) in straw-colored fruit bats in Nigeria, compared with the lower prevalence observed in the present study. In Egypt, PVL-positive strains have been identified in human clinical isolates [74] and raw cow milk samples [75], suggesting that virulent strains may be present across diverse hosts and environments. The phenotypic heterogeneity observed among PVL-positive MRSA isolates, including both *mecA*-positive and *mecA*-negative backgrounds, further reflects the genomic diversity among virulent isolates recovered from bats.

From a One Health perspective, the detection of methicillin-resistant, multidrug-resistant, and PVL-positive *S. aureus* in Egyptian fruit bats is noteworthy and warrants further investigation. This study supports the possibility that bats may serve as ecological carriers of antimicrobial-resistant and virulent *S. aureus* strains, potentially reflecting environmental contamination associated with human and livestock activities in shared habitats. Given their mobility and wide geographic range, bats may represent ecological sentinels worth monitoring within AMR surveillance frameworks.

Several limitations of the present study should be acknowledged. The relatively small sample size limits the precision of prevalence estimates, and larger surveillance studies are needed to obtain more precise estimates of *S. aureus* prevalence and AMR rates in Egyptian fruit bat populations. Importantly, this study was cross-sectional and did not include human or livestock isolates for comparison; therefore, no conclusions regarding zoonotic transmission can be drawn. Additionally, molecular typing approaches such as SCCmec typing, *spa* typing, multilocus sequence typing (MLST), or whole-genome sequencing were beyond the scope of this study. Future investigations incorporating these methods, alongside comparative genomic analyses of bat and human isolates, would provide a more comprehensive understanding of MRSA lineage diversity and potential transmission dynamics involving wildlife reservoirs.

## Conclusion

This study demonstrates that Egyptian fruit bats harbor *S. aureus*, including MRSA, MDR, and PVL-positive strains. The detection of the *mecA* and *pvl* genes in bat-associated isolates indicates the presence of both resistant and virulent strains within bat populations. These findings highlight bats as potential reservoirs of antimicrobial-resistant and virulent *S. aureus* strains, with possible implications for environmental and public health. Furthermore, these findings underscore the need for integrated One Health surveillance encompassing wildlife, humans, and the environment. Continuous monitoring of bat populations can provide valuable insights into wildlife reservoirs of antimicrobial resistance, guiding public health responses and antimicrobial stewardship efforts.

## Data Availability

All the data produced or investigated in this study are included in this published article.
